# The Iron Status of Sickle Cell Anaemia Patients in Ilorin, North Central Nigeria

**DOI:** 10.1155/2015/386451

**Published:** 2015-10-15

**Authors:** Musa A. Sani, James O. Adewuyi, Abiola S. Babatunde, Hannah O. Olawumi, Rasaki O. Shittu

**Affiliations:** ^1^Department of Haematology and Blood Transfusion, Kwara State Specialist Hospital, Sobi, 240001 Ilorin, Nigeria; ^2^Department of Haematology and Blood Transfusion, University of Ilorin Teaching Hospital, PMB 1459, 240003 Ilorin, Nigeria; ^3^Department of Family Medicine, Kwara State Specialist Hospital, Sobi, 240001 Ilorin, Nigeria

## Abstract

*Objectives*. Sickle cell anaemia (SCA) is one of the commonest genetic disorders in the world. It is characterized by anaemia, periodic attacks of thrombotic pain, and chronic systemic organ damage. Recent studies have suggested that individuals with SCA especially from developing countries are more likely to be iron deficient rather than have iron overload. The study aims to determine the iron status of SCA patients in Ilorin, Nigeria. *Methods*. A cross-sectional study of 45 SCA patients in steady state and 45 non-SCA controls was undertaken. FBC, blood film, sFC, sTfR, and sTfR/log sFC index were done on all subjects. *Results*. The mean patients' serum ferritin (589.33 ± 427.61 ng/mL) was significantly higher than the mean serum ferritin of the controls (184.53 ± 119.74 ng/mL). The mean serum transferrin receptor of the patients (4.24 ± 0.17 *μ*g/mL) was higher than that of the controls (3.96 ± 0.17 *μ*g/mL) (*p* = 0.290). The mean serum transferrin receptor (sTfR)/log serum ferritin index of the patients (1.65 ± 0.27 *μ*g/mL) was significantly lower than that of the control (1.82 ± 0.18 *μ*g/mL) (*p* = 0.031). *Conclusion*. Iron deficiency is uncommon in SCA patients and periodic monitoring of the haematological, biochemical, and clinical features for iron status in SCA patients is advised.

## 1. Introduction

Sickle cell anaemia (SCA) is one of the commonest genetic disorders in the world and a major cause of significant morbidity and mortality in Africa [1]. In Nigeria, the incidence of sickle cell anaemia is about 1-2% of the population [1].

The disease consists of a variety of pathological disorders resulting from the inheritance of sickled haemoglobin (HbS) gene either in the homozygous state (SS) or in double heterozygous state with another abnormal haemoglobin gene, for example, SC, SD, S*β*Thal, SO Arab, and SG [2, 3]. The gene abnormality results in the tendency of sickle haemoglobin (HbS) when deoxygenated, to polymerize intracellularly and deform red blood cells into a characteristic sickled shape, thereby producing the clinical manifestation of a chronic haemolytic anaemia with potential iron overload.

However, some studies have shown that iron overload may be a problem only in SCA patients on hypertransfusion programmes [4–6]. Most SCA patients are not hypertransfused and should not have iron overload. On the other hand, some studies have suggested that individuals with sickle cell disease particularly from developing countries, who have never been transfused, are more likely to be iron deficient rather than have iron overload [4]. There is thus some doubt regarding occurrence of iron overload in sickle cell anaemia patients and iron deficiency may be more common than expected especially in men, according to Koduri [7].

Although absence of bone marrow iron remains the gold standard for the diagnosis of iron deficiency, serum ferritin concentration (sFC) adequately reflects iron stores [8–10] and low serum ferritin is highly specific for the diagnosis of iron deficiency. However, sensitivity of serum ferritin may be low in sickle cell anaemia because of nonspecific elevation due to increased red cell turnover [7], chronic inflammation, and the role of serum ferritin as an acute phase reactant [11, 12]. Soluble transferrin receptors (sTfR) are disulfide-linked transmembrane proteins that facilitate the entry of transferrin-bound iron into the cells. It is a truncated monomer of the tissue receptor, lacking the first 100 amino acids (the transmembrane and cytoplasmic domain of the cellular receptor) [13]. The circulating TFRC mirrors the amount of cellular receptors. Measuring the concentration of serum TfR is an alternative method to assess iron status because the concentration increases during iron deficiency. It is thought that the serum TfR concentration is not increased in individuals during an acute phase response; therefore the measurement of serum TfR may help to distinguish between individuals with iron deficiency anaemia and anaemia of chronic disease. Serum TfR concentration is elevated in iron deficiency, haemolytic anaemia, polycythemia, myelodysplastic syndromes, and use of erythropoietic stimulating agents while aplastic anaemia and chronic renal failure result in decrease. Transferrin receptor is a more recent iron marker that is not affected by inflammation [14] and is thought to be a more reliable index of iron status in SCA than serum ferritin. sTfR/sFC index is the ratio of soluble transferrin receptor to log serum ferritin. The use of the log of serum ferritin in this ratio decreases the influence of the acute phase response on the ferritin component of the ratio. The ratio of sTfR/ferritin can be used to quantify the entire spectrum of iron status from positive iron stores through negative iron balance. sTfR reflect the functional iron compartment while TfR-F index takes advantage of the relationship between sTFR and sFC, that is, an increase in TfR and a decrease in the ferritin concentration. sTfR/sFC ratio has been found to be a better reflector of body iron stores [15].

The present study investigated the iron stores in patients with sickle cell anaemia by quantifying both serum transferrin receptor (sTfR) and serum ferritin from which the sTFR/log serum ferritin ratio was computed.

## 2. Subjects

This was a cross-sectional study of sickle cell disease patients attending the Sickle Cell Clinic of University of Ilorin Teaching Hospital, Ilorin, Kwara State, Nigeria. Forty-three confirmed sickle cell anaemia patients attending the clinic were recruited into the study. All subjects were aged 15 years or more and were in a steady state. Forty-three apparently healthy age and sex matched controls with Hb phenotype AA only were recruited from students and patients relatives attending the hospital. Patients were excluded from the study if they have had blood transfusion in the previous 12 weeks or any form of sickle cell crises within 2 weeks of the study. Patients on iron containing haematinics or erythropoiesis stimulating agents, vitamin C, and oral contraceptives were also excluded. So also were patients with history of recent overt blood loss, concurrent medical or surgical conditions like peptic ulcer disease, renal failure, liver disease, malignancy, or chronic inflammatory disease. Ethical clearance was obtained from the Ethical Review and Research Committee of the University of Ilorin Teaching Hospital and written consents for inclusion into the study were obtained from all patients and controls.

## 3. Methods

A structured questionnaire was administered on all subjects recruited for the study and a review of case record folders and routine physical examination were carried out. Venous blood was taken from each subject into a bottle containing K^+^ ethylenediaminetetraacetic acid (EDTA) for estimation of full blood count within 2 hours using Sysmex KX 21N automated cell counter (product of Sysmex Corporation, Tokyo, Japan, with serial number B1786). Blood was also taken into plain specimen bottles and allowed to clot at room temperature to obtain serum. Thin blood film was made from the EDTA sample within 2 hours of collection for microscopic study of red cell morphology. Reticulocyte count preparation was made by the method described by Barbara et al. [16] and the reticulocyte count was expressed as a percentage of cells with blue reticular or granular inclusions over the total red cell counted. From the EDTA sample, Hb genotype was determined by electrophoresis using cellulose acetate membrane at alkaline pH 8.0 as described by Barbara et al. [16]. Serum obtained from the clotted blood sample was used for estimation of serum ferritin concentration (sFC) and transferrin receptors (sTfR) and sTfR/log sFC index was calculated. Quantitative measurement of human soluble transferrin receptor was also done using human sTfR ELISA (enzyme linked immune assay) reagent of Biovendor. Control samples provided were run with duplicates of test sample and OD (optical density) was read at 450 nm and 630 nm. The mean of the difference of the two OD was taken as the OD. Quantitative determination of serum ferritin was done using microwell ferritin enzyme immune assay. Control samples provided were also run with duplicates of test sample and the OD read at 450 nm.

The findings were subjected to statistical analysis in which a *p* value of 0.05 or less was considered as being statistically significant.

## 4. Sample Size

The sample size was calculated from the formula

(1) $$\begin{array}{c} N=\frac{{\left(Z_{1}-X\right)}^{2}\left(P\right)\left(1-P\right)}{D^{2}}, \end{array}$$

where *N* is minimal sample size at 95% confidence level *Z*_1_ − *X* = 1.96 from statistical table, *P* is the best estimate of population, prevalence of SCD obtained from literature 2-3%, and *D* is precision or degree of accuracy which is usually taken as 0.05.

Therefore

(2) $$\begin{array}{c} N=\frac{{\left(1.96\right)}^{2}\times \left(0.025\right)\left(0.97\right)}{{\left(0.05\right)}^{2}}=43. \end{array}$$

## 5. Results

The mean age of the patients was 24.63 ± 9.63 years with a range of 16–60 years. The mean age of the controls was 21.56 ± 6.10 with a range of 16–62 years. All forty-three (43) patients had Hb phenotype S (HbSS). The controls had Hb phenotype AA only. The mean MCV of the patients was 84.95 ± 11.10 femtolitres and 84.67 ± 5.56 femtolitres for the controls, *p* = 0.321 (Table 1). Twenty-eight (65.1%) patients had normocytosis (MCV = 76–96 fL), 10 patients (23.3%) had microcytosis (MCV < 76 fL), and 5 (11.6%) patients had macrocytosis (MCV > 96 fL). In the controls, thirty-eight (88.4%) had normocytosis, 3 (7.0%) had microcytosis, and 2 (4.6%) had macrocytosis. The mean MCH for the patients was 27.49 ± 3.65 picograms while that of the control was 26.48 ± 2.43 picograms. There was no statistically significant difference between these results (*p* value = 0.124). The mean MCHC for the patients was 32.09 ± 2.14 g/dL while that of the control was 31.22 ± 1.30 g/dL. This shows statistically significant difference between these results (*p* value = 0.019). Twenty-three patients (51.1%) had low MCH (MCH < 27 picograms), 16 patients (37.2%) had normal MCH, and 4 (9.3%) had high MCH (MCH > 32 picograms) (Table 2). The mean reticulocyte index in patients was 1.48 ± 1.46% while the mean reticulocyte count in controls was 0.83 ± 0.82. There was statistically significant difference between the patients' reticulocytes index and controls' reticulocyte index, *p* value = 0.001 (Table 1). The mean red cell distribution width (RDW) was 74.64 ± 11.84 and 45.43 ± 3.16 for patients and controls, respectively. There was a significant statistical difference between the mean of the patients and the controls, *p* < 0.001.

Thirty-two (74.4%) patients have had blood transfusion in the past whereas 11 (25.6%) have never had blood transfusion in their lifetime. There was previous history of blood transfusion in only five (11.6%) of the control group. The means of serum ferritin among the nontransfused and transfused patients were 436.54 ± 319.62 ng/mL and 828 ± 452.33 ng/mL, respectively.

The patients' mean serum ferritin was 589.33 ± 427.61 ng/mL and the mean serum ferritin for the controls was 184.53 ± 119.74 ng/mL (Table 3). The mean TfR and mean TfR/sFC index of patients and controls were 4.24 ± 0.17 *μ*g/mL, 1.65 ± 0.27 *μ*g/mL, and 3.96 ± 0.17 *μ*g/mL, 1.82 ± 0.18, respectively (Table 3).

## 6. Discussion

As expected, there were numerical and statistically significant differences in the absolute red cell indices (PCV, Hb, RBC, and RDW) between patients and controls. However, the calculated indices (MCV and MCH) showed no significant differences with the exception of MCHC which confirmed the well-known higher intracellular density of sickle cells.

The bulk of the patients' diet consisted of mainly carbohydrates and only thirty-three of the patients agreed to take protein-containing diet for more than twice daily.

The mean serum ferritin concentration among transfused patients was higher than among the nontransfused patients (*p* value = 0.025). This is in agreement with the study of O'Brien who reported a weak positive correlation (*p* = 0.026) between blood transfusion and ferritin levels of the patients [4]. This is also in keeping with report by Porter and Huehns [17] who obtained a good correlation between serum ferritin and number of blood transfusions.

In this study, twenty-two units of blood was the highest transfusion recorded in a 46-year-old patient which is equivalent to 0.5 units of blood per annum. This observation is in agreement with work of Luzzatto who reported approximately 0.5 units in sickle cell patients [18]. Even the largest number of transfusions does not produce iron overload. There was a linear relationship between age and number of blood transfusions (*r* = 0.505, *p* ≤ 0.001). This was previously observed by O'Brien who found that the concentrations of serum ferritin correlated directly and significantly with age of the patients [4].

In our study the mean patients' serum ferritin (559.33 ± 427.61 ng/mL) was higher than the upper limit of reference range (300 ng/mL) and also statistically significantly (*p* = 0.025) higher when compared with the mean serum ferritin of the controls (184.53 ± 119.74 ng/mL) which was within the reference range. These findings in the patients indicating increase in body iron stores are in keeping with Peterson et al. who reported a high mean serum ferritin in SCD compared to the control [19].

The mean serum transferrin receptor of the patients (4.24 ± 0.17 *μ*g/mL) was not statistically significantly higher than that of the controls (3.96 ± 0.17 *μ*g/mL) (*p* = 0.290).

In this study the reference range of 3.61–4.33 *μ*g/mL was obtained by taking two standard deviations below and above the mean serum transferrin receptor of the controls. Fourteen of our patients had serum transferrin receptor above the study reference range. Transferrin receptor levels are increased during haemolysis which is an expected finding in SCD, thus making it difficult to ascertain the significance of the increase. It has been suggested that serum transferrin receptor seems to be more useful in relation to functional iron compartment than iron stores [20, 21]. It has been further suggested that sTfR in the presence of hypochromia and microcytosis and another parameter, the sTfR/log serum ferritin index may all be diagnostic of iron deficiency in SCD [22]. In this study, the mean serum transferrin receptor (sTfR)/log serum ferritin index of the patients (1.65 ± 0.27 *μ*g/mL) was significantly lower than that of the controls (1.82 ± 0.18 *μ*g/mL) (*p* = 0.031). This is in agreement with Khatami et al. who observed significant differences in sTfR concentration and sTfR/log ferritin (sTfR-F index) in iron deficient groups, compared to thalassemia groups [23]. Also Punnonen and colleague confirmed sTfR/log serum ferritin index as an outstanding parameter for the identification of patient with depleted iron sores [24].

Although various researchers have used different markers to asses iron status in sickle cell disease patients, there is paucity of data on studies that have used sTfR as a marker of iron store in sickle cell disease patients in this region. Researchers in western Nigeria have also reported cases of iron deficiency anaemia in SCD [14, 22, 25, 26]. Williams and Etuk in Eastern Nigeria reported 53% prevalence of iron deficiency among children with SCA [27]. Peterson et al. [19] in his study of iron metabolism in a group of 39 patients with sickle cell disease found iron deficiency anaemia in as high as 28% cases.

Based on microcytosis, hypochromia, and high sTfR/log serum ferritin index, the prevalence of iron deficiency of 7% obtained in this study is much lower than what was obtained by other authors who reported deficiency in 20% and 17.1% of SCD patients using the combination of microcytosis and hypochromia alone [22]. Mohanty et al. in India using elevated ZPP/H ratios as marker reported iron deficiency anaemia in sixty-seven per cent of subjects with sickle cell anaemia [28].

Other researchers in Nigeria and elsewhere have also reported either normal or increased iron stores in their sickle cell disease patients [4, 29–33].

## 7. Conclusion and Recommendations

The results of this study corroborate the findings of previous researchers of normal or high iron stores in SCA patients. On the other hand the prevalence of iron deficiency in sickle cell disease patients in this study was found to be only 7%. Iron deficiency may therefore not be as common as was being reported in some previous studies in sickle cell disease patients. The determination of iron status of SCA patients is better based on measurements of sTfR/log serum ferritin index with or without hypochromia or microcytosis rather than on sFC or hypochromia and microcytosis alone.

There is need to carry out further study on a larger scale on sTfR in this region in order to determine the cutoff value for iron deficiency especially in SCA.

## Figures and Tables

**Table 1:** Red blood cell indices in patients and controls.

Parameter	Patient (number = 45)		Control (number = 45)		*p* value	Remarks
	Mean ± SD	Range	Mean ± SD	Range		
PCV (%)	25.19 ± 4.35	18.40–35.80	40.06 ± 4.07	34.70–46.30	0.018	Significant
Hb conc. (g/dL)	7.81 ± 1.84	5.10–12.80	12.53 ± 1.42	10.60–14.70	<0.001	Significant
RBC (×10^9^/L)	3.00 ± 0.74	1.91–4.94	4.74 ± 0.44	4.80–5.45	0.006	Significant
RETIC index (%)	1.48 ± 1.46	0.19–7.23	0.84 ± 0.82	0.21–4.96	<0.001	Significant
MCV (fL)	84.95 ± 11.10	46.90–110.4	84.69 ± 5.51	75.60–99.30	0.321	Not significant
MCH (pg)	27.49 ± 3.65	20.80–34.40	26.48 ± 2.43	21.90–31.10	0.124	Not significant
MCHC (g/dL)	32.09 ± 2.14	24.10–38.20	31.22 ± 1.30	29.00–33.30	0.019	Significant

PCV: packed cell volume, Hb: haemoglobin concentration, RBC: red blood cell, MCV: mean corpuscular volume, MCH: mean corpuscular haemoglobin, MCHC: mean corpuscular haemoglobin concentration, and RETIC: reticulocyte.

**Table 2:** Prevalence of microcytosis, macrocytosis, hypochromia, hyperchromia, and red cell distribution width in patients and controls.

Indices	Prevalence		Percent	
	Patients	Controls	Patients	Controls
Microcytosis				
MCV <76 fL	10	3	22.2	8.8
Normocytosis				
MCV = 76–96 fL	28	38	66.7	86.7
Macrocytosis				
MCV >96 fL	5	2	11.1	4.4

Total	43.0	43.0	100.0	100.0

Hypochromia				
MCH <27 pg	23	14	53.5	32.6
Normochromia				
MCH = 27–32 pg	16	29	37.2	67.4
Hyperchromia				
MCH >32 pg	4	0	9.3	0

Total	43.0	43.0	100.0	100.0

RDW				
<39.0 fL	0	0	0	0
39–46 fL	2	28	4.4	62.2
>46.0 fL	43	17	95.6	37.8

Total	43.0	43.0	100.0	100.0

MCV: mean corpuscular volume, fL: femtolitre.

MCH: mean corpuscular haemoglobin, pg = picogram.

RDW: red cell distribution width. Normal reference range = 42.5 ± 3.5 fL *p* < 0.001.

**Table 3:** Biochemical parameters in patients and controls.

Parameter	Patients		Control		*p* value
	Mean	SD	Mean	SD	
sFC (ng/mL)	589.33	427.61	184.53	119.74	0.025
TfR (*μ*g/mL)	4.24	0.17	3.96	0.17	0.290
TfR/log sFC index	1.65	0.27	1.82	0.18	0.03

sFC: serum ferritin concentration, TfR: transferrin receptor.
